# The C-reactive protein-to-body mass index ratio predicts prognosis in patients with different types of heart failure

**DOI:** 10.1515/med-2026-1385

**Published:** 2026-03-16

**Authors:** Tao Shi, Jianping Yang, Ningli Zhang, Dan Xu, Fazhi Yang, Sirui Yang, Lixing Chen

**Affiliations:** Department of Cardiology, the First Affiliated Hospital of Kunming Medical University, Kunming, Yunnan, China; College of Big Data, Yunnan Agricultural University, Kunming, Yunnan, China; The Key Laboratory for Crop Production and Smart Agriculture of Yunnan Province, Kunming, Yunnan, China; Department of Anesthesiology, the Second Affiliated Hospital of Kunming Medical University, Kunming, Yunnan, China; Centre of Clinical Research and Education, Curtin School of Population Health, Faculty of Health Sciences, Curtin University, Perth, Australia

**Keywords:** heart failure, C-reactive protein-to-body mass index ratio, ejection fraction, inflammation, nutrition

## Abstract

**Objectives:**

The aim of this study was to explore the predictive value of the C-reactive protein-to-body mass index ratio (CBR) in the prognosis of all-cause mortality in patients with heart failure (HF) with different ejection fractions.

**Methods:**

We included 1196 HF patients from the First Affiliated Hospital of Kunming Medical University after exclusion criteria. Based on the optimal cut-off value from the ROC curves, patients were categorised into low CBR group and high CBR group. The predictive value of the CBR for the prognosis of all-cause mortality in patients with different types of HF was assessed using Kaplan–Meier curves, Cox proportional hazards analyses, cubic spline plots and ROC curves analyses.

**Results:**

Kaplan‒Meier analyses showed that the high CBR group had the highest cumulative incidence of all-cause mortality regardless of the type of HF patients. Multivariate Cox regression analysis revealed that the CBR was an independent predictor of prognosis for all-cause mortality in patients with all types of HF. The cubic spline plots showed a roughly positive association between the CBR and all-cause mortality. The ROC curves showed that for all types of HF patients, the area under the curve for the CBR was the largest relative to individual CRP and BMI.

**Conclusions:**

Regardless of the type of HF patients, the CBR can be a good predictor of prognosis for all-cause mortality in patients with a higher CBR associated with a higher risk of all-cause mortality.

## Introduction

Heart failure (HF) is a serious health concern that affects people around the world and uses up increasing resources for health care in ageing populations [1], [2], [3], [4]. In 2017, the age-standardised prevalence and incidence in China were 1.1 percent and 275 per 100,000 person-years among people aged 25 years or older, respectively, accounting for 12.1 million patients with HF and 3 million patients with incident HF [5]. Increases in HF-related morbidity and mortality have led to an increased focus on HF.

The 2021 ESC Heart Failure Guidelines [6] state that patients with HF can be divided into three categories based on left ventricular ejection fraction (LVEF): HF with reduced ejection fraction (HFrEF) is defined as an LVEF<40 %; HF with mildly reduced ejection fraction (HFmrEF) is defined as an LVEF of 40–49 %; and HF with preserved ejection fraction (HFpEF) is defined as an LVEF≥50 %. There are also differences in risk factors, pathophysiology and treatments for different types of HF [7], 8].

The prognosis of HF patients is significantly impacted by both inflammation and malnutrition. Hepatocytes produce C-reactive protein (CRP), an acute phase reactant that serves as a general indicator of systemic inflammation [9]. CRP has a significant effect on HF patients’ prognosis. Patients with HF have been found to have increased CRP levels in several studies, and rising levels have been linked to greater mortality and morbidity [10], [11], [12]. The body weight index (BMI) is the most commonly used indicator of individual nutritional status, as it is more easily accessible. A cohort study of 7767 HF outpatient patients showed that, compared to patients with a normal BMI, patients with a higher BMI (such as overweight and obese patients) had a lower crude and adjusted risk of all-cause mortality and death from HF [13].

The C-reactive protein-to-body mass index ratio (CBR) integrates both inflammatory (CRP) and nutritional (BMI) dimensions, which are known to significantly impact HF prognosis [11], [14], [15], [16]. While CBR has shown prognostic value in other conditions like cancer [17], its relationship with HF outcomes remains unexplored. We hypothesized that the CBR would serve as an independent predictor of all-cause mortality in patients with HF. Therefore, this study set out to investigate the CBR’s predictive ability for all-cause mortality in patients with different types of HF.

## Methods

### Study population

We included 1221 HF patients who were hospitalised at the First Affiliated Hospital of Kunming Medical University between January 2017 and October 2021 due to acute exacerbation of chronic HF. This study included patients hospitalised for HF who were New York Heart Association (NYHA) functional class III or IV and had brain natriuretic peptide (BNP) levels above 500 pg/mL. After excluding patients who lacked necessary data (CRP, BMI), had severe comorbidities (malignant tumours, infectious diseases, haematological diseases, or severe kidney or liver dysfunction), and were lost to follow-up, 1,196 patients were ultimately included in this study. Among them, there were 499 patients with HFrEF, 233 patients with HFmrEF, and 464 patients with HFpEF.

### Data collection

At admission, blood samples, electrocardiograms, cardiac ultrasound data, and demographic and clinical information were gathered. Data included patient age, sex, BMI, heart rate, NYHA class, smoking, blood pressure (BP), troponin, white blood cell (WBC), neutrophils, lymphocytes, red blood cell (RBC), CRP, haemoglobin, platelets, albumin, BNP, potassium, sodium, chlorine, aminotransferase (ALT), aspartate aminotransferase (AST), uric acid, estimated glomerular filtration rate (eGFR), HF type, QRS width, left atrial diameter (LAd), left ventricular end-diastolic diameter (LVDd), right atrial diameter (RAd), right ventricular diameter (RVd), medical history, and treatment history. Following an 8–12 h overnight fast, blood was collected from each patient and submitted to the laboratory of the First Affiliated Hospital of Kunming Medical University’s laboratory.

CBR refers to the ratio of CRP to BMI, where BMI=weight in kilograms/(height in metres)^2^ and CRP=standard C-reactive protein in milligrams per litre.

The researchers gathered the survivorship data through phone conversations with the patients or their relatives. All-cause mortality served as the study’s primary outcome.

### Statistical analysis

On the basis of LVEF, patients were categorised into HFrEF+HFmrEF (LVEF<50 %) and HFpEF (LVEF≥50 %) groups to assess the consistency of CBR’s prognostic value across different HF types, given the known heterogeneity in their clinical profiles. Subsequently, according to the optimal cut-off values derived from the CBR ROC curves, patients were categorised into G1a and G2a groups for all patients, G1r and G2r groups for HFrEF+HFmrEF patients, and G1p and G2p groups for HFpEF patients. The mean±standard deviation was used to represent normally distributed continuous variables, whereas the median with interquartile range was used to express skewed continuous variables. Numbers and percentages were used to represent the category variables. For BNP with a severe skewness distribution, logarithmic conversion was performed. The independent sample t tests were used to compare continuous variables that were normally distributed between groups, and Mann‒Whitney U tests were used to compare continuous variables that were skewed between groups. The chi-square test was used to compare categorical variables. The Kaplan‒Meier method was used to estimate the probability of all-cause mortality, and the log-rank test was used to evaluate discrepancies in the curves. The effect of the CBR on all-cause mortality in HF patients was evaluated using univariate and multivariate Cox proportional hazards models. We selected variables for inclusion in Cox proportional hazards models based on clinical relevance, literature-derived prognostic factors, and univariate screening (p<0.05). To address potential time bias due to the extended enrolment period, we performed sensitivity analyses by restricting the follow-up duration to fixed periods of 1 year and 3 years from the date of enrolment for all patients. The cubic spline models showed the relationship between the CBR and the risk of all-cause mortality in the fully adjusted model. To assess the predictive usefulness of the CBR for all-cause mortality in HF patients, we performed receiver operating characteristic (ROC) curve analysis. To internally validate the robustness of our Cox proportional hazards models and correct for potential over-optimism, we performed bootstrapping with 1,000 resamples. The optimism-corrected concordance index (C-index) was calculated to provide a more robust estimate of the model’s discriminative ability. In all analyses, a two-tailed p value of less than 0.05 was considered indicative of statistical significance. R 4.3.1 and IBM SPSS Statistics 26.0 were used for all data analysis in this study.

### Ethics approval

The First Affiliated Hospital of Kunming Medical University’s Medical Ethics Committee gave its approval for the study, which was carried out in compliance with the Declaration of Helsinki. The study’s ethics number was Ethics L No. 173 (2022).

## Results

### Baseline patient characteristics

This study included 1,196 patients with HF. Based on LVEF, we divided the patients into two groups: the HFrEF+HFmrEF group (LVEF<50 %, n=732) and the HFpEF group (LVEF≥50 %, n=464). Compared with patients with HFpEF, those with HFrEF+HFmrEF exhibited higher proportions of male, NYHA class III/IV, smoking, hypertension, diabetes, atrial fibrillation, and stroke; higher levels of lgBNP, RBC, haemoglobin, albumin, ALT, AST, uric acid, and eGFR; larger QRS wave, LAd, LVDd, and RVd; and lower age and systolic BP. (p<0.05). The specific baseline comparison details are shown in Table 1.

**Table 1:** Baseline characteristics according to the left ventricular ejection fraction (LVEF).

Variables	Total (n=1,196)	Left ventricular ejection fraction groups		p-Value
		HFrEF+HFmrEF (LVEF<50 %, n=732)	HFpEF (LVEF≥50 %, n=464)	
**Basic characteristics**				
Follow-up time, days	755 (346, 1,119)	737 (333, 1,102)	768 (358, 1,138)	0.694
Age, years	66.83 ± 12.52	65.23 ± 12.53	69.36 ± 12.10	<0.001
Male	742 (62.0 %)	478 (65.3 %)	264 (56.9 %)	0.004
BMI, kg/m2	23.02 ± 3.81	22.95 ± 3.77	23.12 ± 3.88	0.450
Heart rate (beats/minute)	85.24 ± 21.02	85.84 ± 20.09	84.29 ± 22.38	0.214
NYHA class				0.006
III	752 (62.9 %)	438 (59.8 %)	314 (67.7 %)	
IV	444 (37.1 %)	294 (40.2 %)	150 (32.3 %)	
Systolic BP, mmHg	122.10 ± 22.94	119.85 ± 22.01	125.64 ± 23.94	<0.001
Diastolic BP, mmHg	76.23 ± 15.04	76.77 ± 14.83	75.39 ± 15.34	0.123
Smoking	409 (34.2 %)	272 (37.2 %)	137 (29.5 %)	0.007
**Laboratory indicators**				
LgBNP, pg/mL	3.17 ± 0.28	3.25 ± 0.26	3.04 ± 0.27	<0.001
Troponin, ng/mL	0.05 (0.03, 0.12)	0.05 (0.03, 0.11)	0.05 (0.03, 0.16)	0.141
CRP, mg/L	7.47 (3.00, 21.78)	7.80 (3.01, 21.69)	7.13 (2.99, 22.25)	0.639
WBC (10ˆ9/L)	6.93 (5.54, 9.07)	6.80 (5.50, 8.76)	7.16 (5.62, 9.39)	0.070
Neutrophil (10ˆ9/L)	4.52 (3.49, 6.50)	4.44 (3.45, 6.24)	4.68 (3.53, 6.78)	0.061
Lymphocyte (10ˆ9/L)	1.38 (1.02, 1.83)	1.43 (1.05, 1.85)	1.35 (0.98, 1.80)	0.184
RBC (10ˆ12/L)	4.54 ± 0.77	4.61 ± 0.74	4.45 ± 0.79	0.001
Haemoglobin, g/L	138.11 ± 24.17	140.29 ± 22.67	134.66 ± 26.02	<0.001
Platelet (10ˆ9/L)	192.00 (148.00, 243.00)	189.00 (146.25, 240.00)	197.50 (152.00, 248.00)	0.105
Potassium, mmol/L	3.94 ± 0.60	3.96 ± 0.58	3.92 ± 0.64	0.253
Sodium, mmol/L	141.01 ± 4.43	140.89 ± 4.30	141.19 ± 4.63	0.249
Chlorine, mmol/L	102.92 ± 4.68	102.78 ± 4.46	103.14 ± 4.99	0.188
Albumin, g/L	36.69 ± 4.55	37.11 ± 4.34	36.04 ± 4.80	<0.001
ALT, IU/L	25.05 (16.70, 43.33)	26.50 (17.23, 46.30)	23.35 (15.15, 38.00)	<0.001
AST, IU/L	28.85 (20.58, 42.30)	29.55 (21.30, 46.25)	26.55 (19.08, 40.38)	<0.001
Uric acid, umol/L	477.70 (371.28, 588.35)	508.30 (406.50, 620.50)	433.60 (341.75, 544.10)	<0.001
eGFR, mL/min	44.08 (32.32, 56.67)	45.59 (33.33, 56.91)	41.71 (31.10, 56.15)	0.017
CBR	0.34 (0.13, 0.99)	0.37 (0.13, 1.00)	0.32 (0.13, 0.98)	0.635
**ECG parameters and cardiac ultrasound index**				
QRS wave, ms	106.00 (94.00, 128.00)	112.00 (98.00, 136.00)	100.00 (90.00, 116.00)	<0.001
LAd, mm	42.37 ± 9.38	43.57 ± 8.98	40.48 ± 9.69	<0.001
LVDd, mm	56.31 ± 12.65	61.46 ± 11.31	48.16 ± 10.14	<0.001
RAd, mm	51.97 ± 12.54	52.26 ± 12.28	51.52 ± 12.94	0.324
RVd, mm	67.37 ± 16.59	68.66 ± 17.47	65.30 ± 14.84	0.001
**Medical history**				
Coronary artery disease	619 (51.8 %)	378 (51.6 %)	241 (51.9 %)	0.919
Hypertension	660 (55.2 %)	364 (49.7 %)	296 (63.8 %)	<0.001
Diabetes	341 (28.5 %)	191 (26.1 %)	150 (32.3 %)	0.020
Atrial fibrillation	406 (33.9 %)	214 (29.2 %)	192 (41.4 %)	<0.001
Stroke	167 (14.0 %)	90 (12.3 %)	77 (16.6 %)	0.037
**Treatment**				
SGLT-2I	270 (22.6 %)	155 (21.2 %)	115 (24.8 %)	0.146
β-receptor blockers	840 (70.2 %)	522 (71.3 %)	318 (68.5 %)	0.306
Diuretics	943 (78.8 %)	554 (75.7 %)	389 (83.8 %)	0.001
ACEI/ARB/ARNI	671 (56.1 %)	383 (52.3 %)	288 (62.1 %)	0.001
CRT/CRTD	116 (9.7 %)	80 (10.9 %)	36 (7.8 %)	0.071

① Differences in normally distributed continuous variables were compared using independent sample t tests, and those in nonnormally distributed data were compared using Mann‒Whitney U tests. Chi-square tests were used to compare differences in categorical variables between groups. p values were derived from comparing HFrEF+HFmrEF group and HFpEF group. p<0.05 was considered indicative of statistical significance. ② HFrEF, heart failure with reduced ejection fraction; HFmrEF, heart failure with mildly reduced ejection fraction; HFpEF, heart failure with preserved ejection fraction; BMI, body mass index; NYHA, New York Heart Association; BP, blood pressure; BNP, brain natriuretic peptide; CRP, C-reactive protein; WBC, white blood cell; RBC, red blood cell; ALT, alanine aminotransferase; AST, aspartate aminotransferase; eGFR, estimated glomerular filtration rate; CBR, C-reactive protein-to-body mass index ratio; LAd, left atrium diameter; LVDd, left ventricular end-diastolic diameter; RAd, right atrium diameter; RVd, right ventricle diameter; SGLT-2I, sodium-glucose cotransporter 2 inhibitor; ACEI, angiotensin converting enzyme inhibitor; ARB, angiotensin II receptor blocker; ARNI, angiotensin receptor-enkephalinase inhibitor; CRT, cardiac resynchronisation therapy; CRTD, cardiac resynchronisation therapy defibrillator.

### C-reactive protein-to-body mass index ratio (CBR) and all-cause mortality

**Figure 1:**
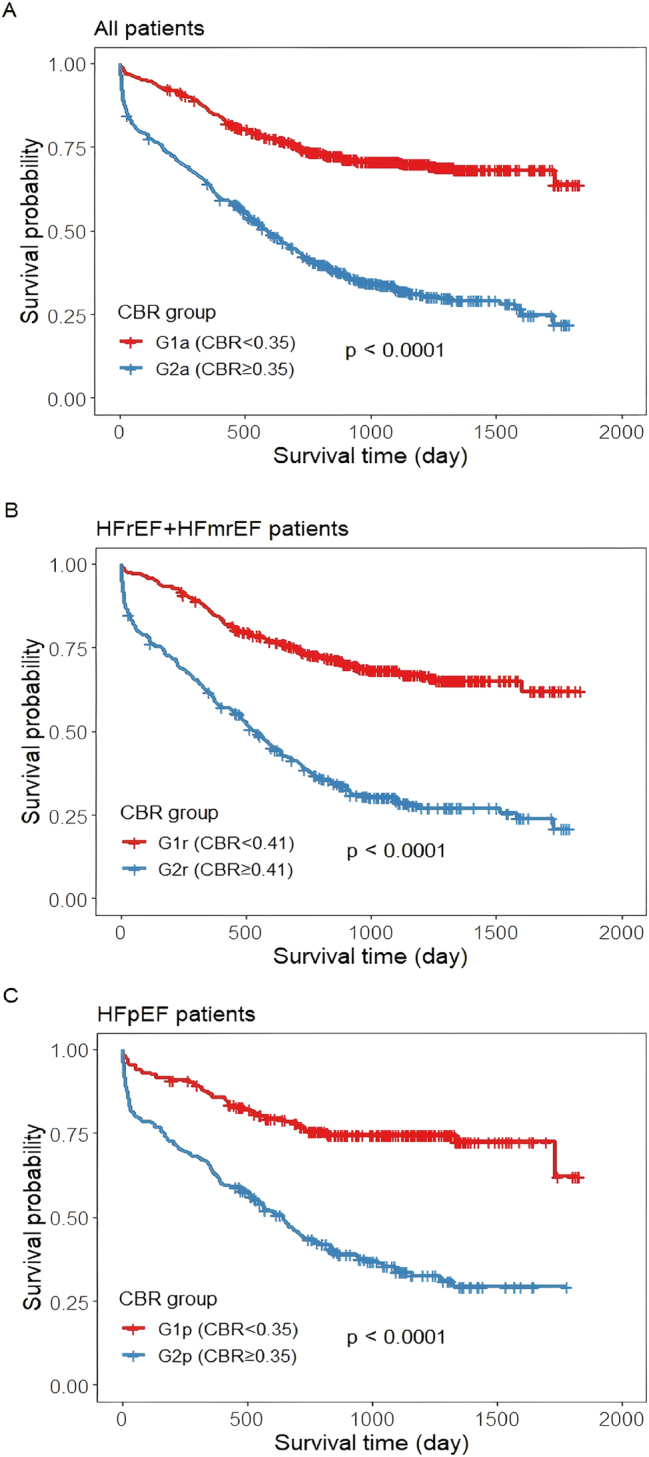
Kaplan‒Meier survival curves for all patients (A), HFrEF+HFmrEF patients (B) and HFpEF patients (C) across the C-reactive protein-to-body mass index ratio (CBR).

During a median follow-up of 755 days after discharge, a total of 492 patients (41.1 %) died. Based on the optimal cut-off value from the ROC curves for CBR, all patients were divided into the G1a group (CBR<0.35, n=599) and the G2a group (CBR≥0.35, n=597); patients with HFrEF+HFmrEF were divided into the G1r group (CBR<0.41, n=379) and the G2r group (CBR≥0.41, n=353); and patients with HFpEF were divided into the G1p group (CBR<0.35, n=244) and the G2p group (CBR≥0.35, n=220) (Table 2). To test the prognostic value of the CBR in patients with different types of HF, we implemented a Kaplan‒Meier analysis. Kaplan‒Meier analysis showed that the cumulative incidence of all-cause mortality was always highest for high CBR level groups (G2a, G2r, G2p) and lowest for low CBR level groups (G1a, G1r, G1p), whether for all patients, HFrEF+HFmrEF patients or HFpEF patients (all patients: log-rank test, p<0.0001; HFrEF+HFmrEF patients: log-rank test, p<0.0001; HFpEF patients: log-rank test, p<0.0001) (Figure 1).

**Table 2:** The time-dependent ROC curves’ AUC (A) and corresponding grouping (B) for the C-reactive protein-to-body mass index ratio (CBR) in heart failure with different ejection fractions.

A	AUC, cutoff value, sensitivity and specificity of the CBR			
	AUC (95 % CI)	Cutoff value	Sensitivity	Specificity
All (n=1,196)	0.737 (0.701, 0.761)	0.35	69.4 %	68.1 %
HFrEF+HFmrEF (n=732)	0.741 (0.706, 0.771)	0.41	67.2 %	70.7 %
HFpEF (n=464)	0.732 (0.690, 0.772)	0.35	69.5 %	69.7 %

**B**	**CBR grouping based on cutoff values**			

All (n=1,196)	G1a (CBR<0.35, n=599)		G2a (CBR≥0.35, n=597)	
HFrEF+HFmrEF (n=732)	G1r (CBR<0.41, n=379)		G2r (CBR≥0.41, n=353)	
HFpEF (n=464)	G1p (CBR<0.35, n=244)		G2p (CBR≥0.35, n=220)	

The n denotes the number of patients. AUC, area under the curve; ROC, receiver operating characteristic; CI, confidence interval.

### C-reactive protein-to-body mass index ratio (CBR) as a predictor of all-cause mortality

CBR was a significant predictor of all-cause mortality in all patients, HFrEF+HFmrEF patients and HFpEF patients (all patients: HR 1.246, 95 % CI 1.204–1.290, p<0.001; HFrEF+HFmrEF patients: HR 1.247, 95 % CI 1.186–1.312, p<0.001; HFpEF patients: HR 1.243, 95 % CI 1.184–1.305, p<0.001) (Table 3A Unadjusted). After adjustment for age, sex, NYHA class, heart rate and smoking, the CBR was an independent predictor of all-cause mortality (all patients: HR 1.224, 95 % CI 1.179–1.270, p<0.001; HFrEF+HFmrEF patients: HR 1.247, 95 % CI 1.183–1.316, p<0.001; HFpEF patients: HR 1.193, 95 % CI 1.128–1.262, p<0.001) (Table 3A, Model 2). With further adjustments for lgBNP, troponin, WBC, neutrophil, lymphocyte, serum sodium, serum chlorine, albumin, uric acid, eGFR, coronary artery disease (CAD), hypertension, diabetes, atrial fibrillation, stroke, SGLT-2I, β-receptor blockers and ACEI/ARB/ARNI, the CBR continued to be independently associated with all-cause mortality (all patients: HR 1.167, 95 % CI 1.114–1.222, p<0.001; HFrEF+HFmrEF patients: HR 1.149, 95 % CI 1.077–1.226, p<0.001; HFpEF patients: HR 1.159, 95 % CI 1.073–1.252, p<0.001) (Table 3A, Model 4).

**Table 3:** Cox proportional hazard models for the association of the C-reactive protein-to-body mass index ratio (CBR) and the risk of mortality. The CBR as a continuous variable (A); the CBR as a categorical variable (B).

A	CBR as a continuous variable^a^					
Model	All (n=1,196)		HFrEF+HFmrEF (n=732)		HFpEF (n=464)	
	HR (95 % CI)	p-Value	HR (95 % CI)	p-Value	HR (95 % CI)	p-Value
Unadjusted	1.246 (1.204, 1.290)	<0.001	1.247 (1.186, 1.312)	<0.001	1.243 (1.184, 1.305)	<0.001
Model 1	1.234 (1.192, 1.278)	<0.001	1.241 (1.179, 1.305)	<0.001	1.224 (1.164, 1.286)	<0.001
Model 2	1.224 (1.179, 1.270)	<0.001	1.247 (1.183, 1.316)	<0.001	1.193 (1.128, 1.262)	<0.001
Model 3	1.165 (1.113, 1.220)	<0.001	1.149 (1.078, 1.224)	<0.001	1.155 (1.071, 1.246)	<0.001
Model 4	1.167 (1.114, 1.222)	<0.001	1.149 (1.077, 1.226)	<0.001	1.159 (1.073, 1.252)	<0.001

B		CBR as a categorical variable^b^								
	Unadjusted		Model 1		Model 2		Model 3		Model 4	
	HR (95 % CI)	p-Value	HR (95 % CI)	p-Value	HR (95 % CI)	p-Value	HR (95 % CI)	p-Value	HR (95 % CI)	p-Value
**All (n=1,196)**										
G1a	reference		reference		reference		reference		reference	
G2a	2.892 (2.435, 3.435)	<0.001	2.762 (2.325, 3.283)	<0.001	2.632 (2.210, 3.135)	<0.001	1.959 (1.622, 2.367)	<0.001	1.942 (1.607, 2.347)	<0.001
**HFrEF+HFmrEF (n=732)**										
G1r	reference		reference		reference		reference		reference	
G2r	3.163 (2.537, 3.942)	<0.001	3.033 (2.431, 3.784)	<0.001	2.939 (2.349, 3.678)	<0.001	2.273 (1.783, 2.896)	<0.001	2.227 (1.744, 2.845)	<0.001
**HFpEF (n=464)**										
G1p	reference		reference		reference		reference		reference	
G2p	3.397 (2.510, 4.597)	<0.001	3.053 (2.254, 4.136)	<0.001	2.808 (2.066, 3.817)	<0.001	2.156 (1.553, 2.994)	0.001	2.127 (1.527, 2.964)	0.001

Model 1: adjusted for age and sex. Model 2: adjusted for Model 1 + NYHA class, heart rate and smoking. Model 3: adjusted for Model 2 + lgBNP, troponin; WBC, neutrophil, lymphocyte, serum sodium, serum chlorine, albumin, uric acid and eGFR. Model 4: adjusted for Model 3 + CAD, hypertension, diabetes, atrial fibrillation, stroke, SGLT-2I, β-receptor blockers and ACEI/ARB/ARNI. HR, hazard ratio; CI, confidence interval; NYHA, New York Heart Association; BNP, brain natriuretic peptide; WBC, white blood cell; eGFR, estimated glomerular filtration rate; CAD, coronary artery disease; SGLT-2I, sodium-glucose cotransporter 2 inhibitor; ACEI, angiotensin converting enzyme inhibitor; ARB, angiotensin II receptor blocker; ARNI, angiotensin receptor-enkephalinase inhibitor. G1a (CBR<0.35), G2a (CBR≥0.35); G1r (CBR<0.41), G2r (CBR≥0.41); G1p (CBR<0.35), G2p (CBR≥0.35). ^a^The reported HRs represent a 1-unit increase of the CBR. ^b^The reported HRs were derived using G1a (CBR<0.35), G1r (CBR<0.41) and G1p (CBR<0.35) as reference.

We used CBR as a categorical variable. After Model 4 correction, using G1a as a reference, for all patients, the risk of death was 1.942 times higher for G2a than for G1a; using G1r as a reference, for HFrEF+HFmrEF patients, the risk of death was 2.227 times higher for G2r than for G1r; using G1p as a reference, for HFpEF patients, the risk of death at G2p was 2.127 times that of G1p (Table 3B). In conclusion, the risk of all-cause mortality was lowest for the low CBR level groups (G1a, G1r, G1p) and highest for the high CBR level groups (G2a, G2r, G2p), regardless of whether the analysis included all patients, HFrEF+HFmrEF patients, or HFpEF patients.

To address the potential for time bias arising from the extended enrolment period (2017–2021), we performed sensitivity analyses by administratively censoring follow-up at 1 year and 3 years post-enrolment. In the 1-year fixed follow-up analysis, the CBR remained a significant independent predictor of all-cause mortality after full adjustment (all patients: HR 1.133, 95 % CI 1.067–1.203, p<0.001; HFrEF+HFmrEF patients: HR 1.106, 95 % CI 1.011–1.211, p=0.028; HFpEF patients: HR 1.149, 95 % CI 1.047–1.261, p=0.003) (Table 4). Similarly, in the 3-year fixed follow-up analysis, the fully adjusted HR for CBR was 1.165 (95 % CI 1.111–1.221, p<0.001) for all patients, 1.145 (95 % CI 1.071–1.223, p<0.001) for HFrEF+HFmrEF patients, and 1.156 (95 % CI 1.069–1.250, p<0.001) for HFpEF patients (Table 4). These results confirm that the prognostic value of CBR is robust and not materially confounded by differences in enrolment time.

**Table 4:** Sensitivity analyses of the association between the CBR and All-cause mortality using fixed follow-up periods of 1 and 3 years.

Patient group	Fixed follow-up	Events N	Adjusted HR (95 % CI)	p-Value
All patients (n=1,196)	Original	563	1.167 (1.114, 1.222)	<0.001
	1-Year	310	1.133 (1.067, 1.203)	<0.001
	3-Year	543	1.165 (1.111, 1.221)	<0.001
HFrEF+HFmrEF patients (n=732)	Original	363	1.149 (1.077, 1.226)	<0.001
	1-Year	195	1.106 (1.011, 1.211)	0.028
	3-Year	349	1.145 (1.071, 1.223)	<0.001
HFpEF patients (n=464)	Original	200	1.159 (1.073, 1.252)	<0.001
	1-Year	115	1.149 (1.047, 1.261)	0.003
	3-Year	194	1.156 (1.069, 1.250)	<0.001

The reported HRs represent a 1-unit increase of the CBR. All models are adjusted for covariates in Model 4 (age, sex, NYHA class, heart rate, smoking; lgBNP, troponin; WBC, neutrophil, lymphocyte, serum sodium, serum chlorine, albumin, uric acid; eGFR, CAD, hypertension, diabetes, atrial fibrillation, stroke, SGLT-2I, β-receptor blockers, ACEI/ARB/ARNI). HR, hazard ratio; CI, confidence interval; NYHA, New York Heart Association; BNP, brain natriuretic peptide; WBC, white blood cell; eGFR, estimated glomerular filtration rate; CAD, coronary artery disease; SGLT-2I, sodium-glucose cotransporter 2 inhibitor; ACEI, angiotensin converting enzyme inhibitor; ARB, angiotensin II receptor blocker; ARNI, angiotensin receptor-enkephalinase inhibitor.

To assess whether the prognostic value of CBR remains consistent across different types of HF, we conducted an interaction analysis. Interaction tests revealed no significant effect modification by HF type (p for interaction=0.696), supporting the consistent prognostic role of CBR across all HF phenotypes.

**Figure 2:**
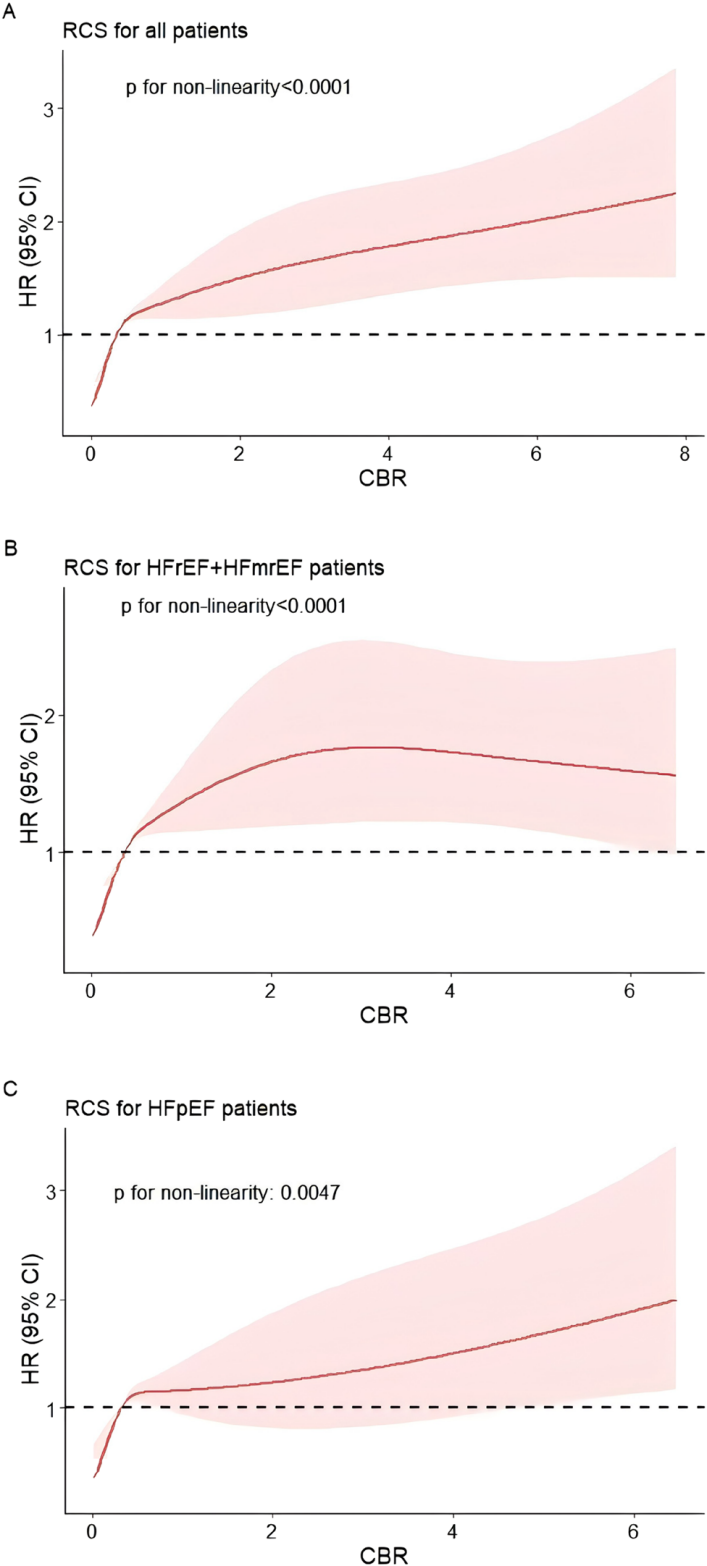
Cubic spline plots for the associations of the C-reactive protein-to-body mass index ratio (CBR) with the risk of all-cause mortality in all patients (A), HFrEF+HFmrEF patients (B) and HFpEF patients (C). HRs (solid line) and 95 % CIs (dashed line) were estimated in a Cox proportional hazards model with adjustment for age, sex, NYHA class, heart rate, smoking, lgBNP, troponin, WBC, neutrophil, lymphocyte, serum sodium, serum chlorine, albumin, uric acid, eGFR, CAD, hypertension, diabetes, atrial fibrillation, stroke, SGLT-2I, β-receptor blockers and ACEI/ARB/ARNI. HR, hazard ratio; CI, confidence interval; NYHA, New York Heart Association; BNP, brain natriuretic peptide; WBC, white blood cell; eGFR, estimated glomerular filtration rate; CAD, coronary artery disease; SGLT-2I, sodium-glucose cotransporter 2 inhibitor; ACEI, angiotensin converting enzyme inhibitor; ARB, angiotensin II receptor blocker; ARNI, angiotensin receptor-enkephalinase inhibitor.

After Model 4 correction, the cubic spline plots showed a roughly positive association between the CBR and all-cause mortality, whether in all patients, HFrEF+HFmrEF patients or HFpEF patients (Figure 2). As the CBR value increased, the patient’s risk of all-cause mortality also increased, albeit not by the same magnitude, but in a roughly upwards trend.

### Predictive ability of the C-reactive protein-to-body mass index ratio (CBR) in patients with heart failure

**Figure 3:**
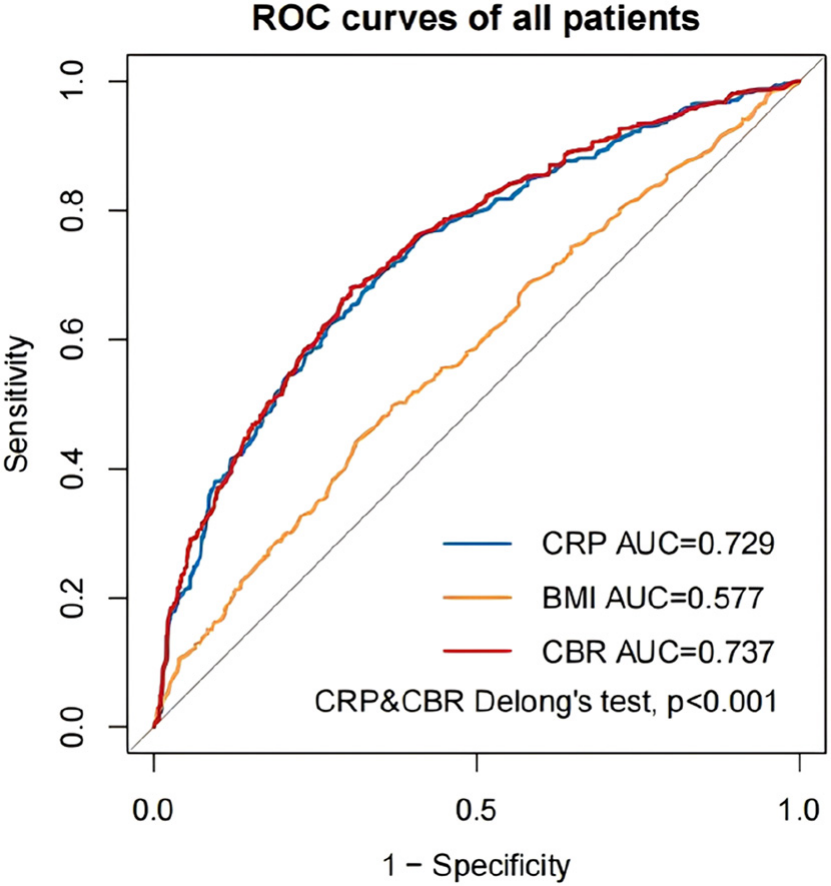
Time-dependent receiver operating characteristic (ROC) curves of C-reactive protein (CRP), body mass index (BMI) and C-reactive protein-to-body mass index ratio (CBR) with the reference line for all-cause mortality in all patients. AUC, area under the curve.

**Figure 4:**
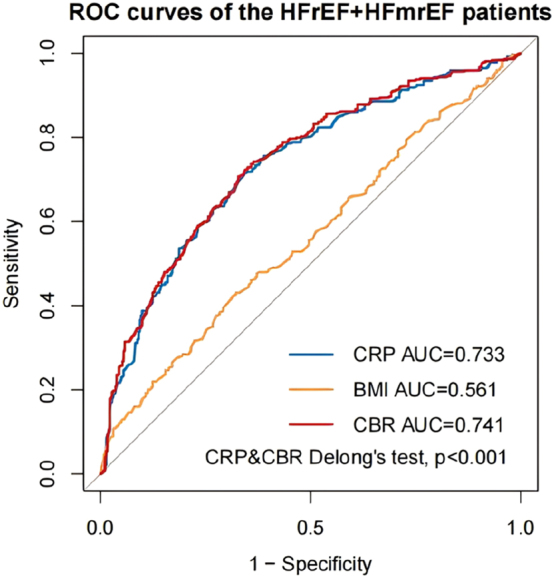
Time-dependent receiver operating characteristic (ROC) curves of C-reactive protein (CRP), body mass index (BMI) and C-reactive protein-to-body mass index ratio (CBR) with the reference line for all-cause mortality in HFrEF+HFmrEF patients. AUC, area under the curve.

**Figure 5:**
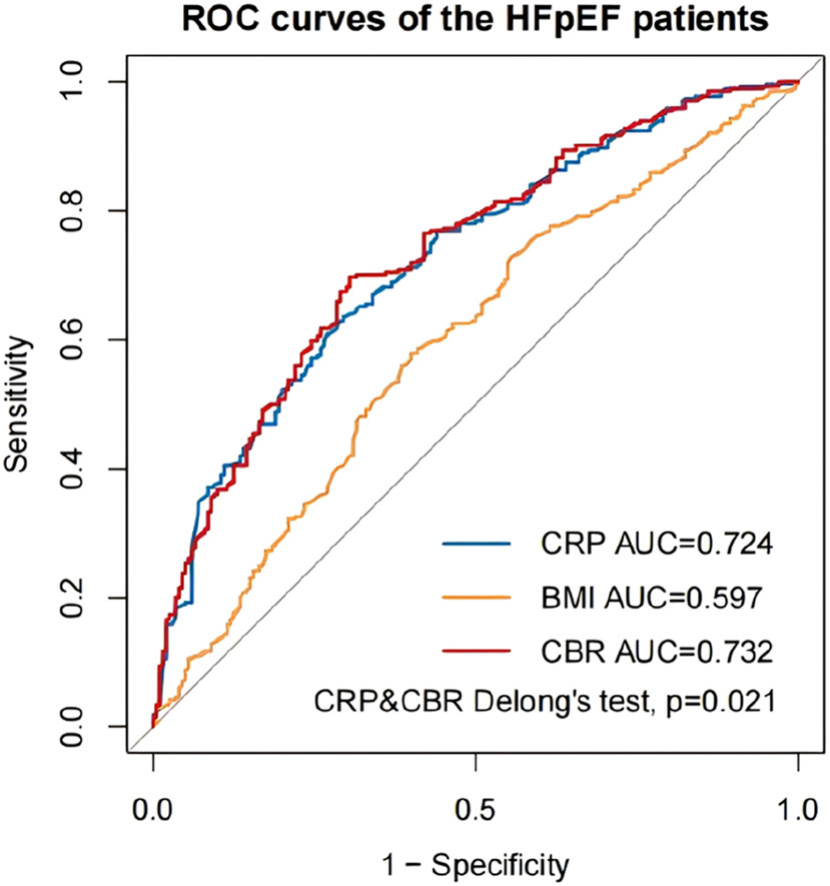
Time-dependent receiver operating characteristic (ROC) curves of C-reactive protein (CRP), body mass index (BMI) and C-reactive protein-to-body mass index ratio (CBR) with the reference line for all-cause mortality in HFpEF patients. AUC, area under the curve.

We constructed time-dependent ROC curves to investigate the CBR’s ability to predict all-cause mortality in HF patients. In all patients, the AUC for CRP was 0.729, the AUC for BMI was 0.577, and the AUC for the CBR was 0.737, with the AUC for the CBR being the largest (Delong’s test, p<0.001) (Figure 3). In HFrEF+HFmrEF patients, the AUC for CRP was 0.733, the AUC for BMI was 0.561, and the AUC for the CBR was 0.741, with the AUC for the CBR being the largest (Delong’s test, p<0.001) (Figure 4). In HFpEF patients, the AUC for CRP was 0.724, the AUC for BMI was 0.597, and the AUC for the CBR was 0.732, with the AUC for the CBR being the largest (Delong’s test, p=0.021) (Figure 5). In summary, we can conclude that the CBR is superior to CRP and BMI in predicting all-cause mortality in HF patients, whether for all patients, HFrEF+HFmrEF patients, or HFpEF patients. In addition, we compared the AUC of CBR with the AUC of other common prognostic indicators (age, heart rate, WBC, neutrophil, lymphocyte, lgBNP, potassium, sodium, chlorine, uric acid, eGFR) of HF (Table 5). Using Delong’s test, we can conclude that CBR is a better predictor of prognosis in HF patients than most common indicators.

**Table 5:** Area under the curve (AUC) of the time-dependent receiver operating characteristic (ROC) curves for C-reactive protein-to-body mass index ratio (CBR) and other prognostic indicators of heart failure.

Variable	All (n=1,196)		HFrEF+HFmrEF (n=732)		HFpEF (n=464)	
	AUC (95 % CI)	p-Value^a^	AUC	p-Value^a^	AUC	p-Value^a^
CBR	0.737 (0.701, 0.761)	–	0.741 (0.706, 0.771)	–	0.732 (0.690, 0.772)	–
Age	0.623 (0.595, 0.650)	<0.001	0.604 (0.567, 0.639)	<0.001	0.680 (0.635, 0.722)	0.119
Heart rate	0.552 (0.524, 0.581)	<0.001	0.560 (0.523, 0.596)	<0.001	0.537 (0.490, 0.583)	<0.001
WBC	0.549 (0.520, 0.577)	<0.001	0.558 (0.522, 0.595)	<0.001	0.539 (0.493, 0.585)	<0.001
Neutrophil	0.566 (0.538, 0.595)	<0.001	0.578 (0.541, 0.614)	<0.001	0.556 (0.509, 0.602)	<0.001
Lymphocyte	0.599 (0.571, 0.627)	<0.001	0.591 (0.554, 0.627)	<0.001	0.618 (0.572, 0.662)	<0.001
LgBNP	0.643 (0.615, 0.671)	<0.001	0.646 (0.610, 0.681)	<0.001	0.625 (0.579, 0.670)	0.002
Potassium	0.528 (0.499, 0.557)	<0.001	0.535 (0.498, 0.573)	<0.001	0.515 (0.468, 0.562)	<0.001
Sodium	0.607 (0.578, 0.635)	<0.001	0.605 (0.568, 0.641)	<0.001	0.608 (0.562, 0.654)	<0.001
Chlorine	0.623 (0.594, 0.651)	<0.001	0.617 (0.580, 0.653)	<0.001	0.627 (0.581, 0.672)	0.001
Uric acid	0.586 (0.557, 0.615)	<0.001	0.597 (0.560, 0.633)	<0.001	0.555 (0.508, 0.601)	<0.001
eGFR	0.650 (0.622, 0.677)	<0.001	0.652 (0.616, 0.686)	<0.001	0.659 (0.614, 0.702)	0.028

^a^p values were derived by comparing the AUCs of the CBR with other indicators using Delong’s test. p<0.05 was considered indicative of statistical significance. CI, confidence interval; WBC, white blood cell; BNP, brain natriuretic peptide; eGFR, estimated glomerular filtration rate.

To account for potential overfitting and internally validate the robustness of the Cox proportional hazards model (Model 4), we calculated the optimism-corrected C-index via bootstrapping. The corrected C-index for the CBR model was 0.734 in all patients, 0.709 in the HFrEF+HFmrEF patients, and 0.748 in the HFpEF patients. These outstanding results (all C-index >0.70) confirm the robust predictive performance of the CBR model across the HF spectrum, with powerful performance in HFpEF patients.

## Discussion

In the present study, we found that the CBR is a robust and independent predictor of all-cause mortality in patients with HF, irrespective of ejection fraction subtype. Specifically, a higher CBR was consistently associated with an increased risk of death across all patient groups (all patients, HFrEF+HFmrEF, and HFpEF). According to this study, the CBR was a significant factor in predicting the prognosis of patients with different types of HF. Using univariate and multivariate Cox proportional hazards analyses, we found that the CBR was an independent predictor of all-cause mortality in patients with all types of HF. In the fully adjusted model, when CBR was used as a categorical variable, it was shown that the risk of all-cause mortality was lowest for the low CBR level groups (G1a: CBR<0.35; G1r: CBR<0.41; G1p: CBR<0.35) and highest for the high CBR level groups (G2a: CBR≥0.35; G2r: CBR≥0.41; G2p: CBR≥0.35), whether for all patients, HFrEF+HFmrEF patients, or HFpEF patients. Cubic spline plots showed a roughly positive correlation between the CBR and all-cause mortality in all patients, HFrEF+HFmrEF patients, and HFpEF patients. Our study also suggested that the AUC of the CBR was always greater than the AUC of CRP and BMI, regardless of the type of HF. The bootstrapping internal validation of our Cox proportional hazards models revealed outstanding predictive performance that was well maintained after accounting for potential overfitting (optimism-corrected C-index: 0.709–0.748). The excellent discriminative ability across all patients, HFrEF+HFmrEF patients, and HFpEF patients provides strong evidence for the robustness of our findings. In conclusion, the CBR can be a good predictor of prognosis in patients with HF.

The CBR is the ratio of CRP to BMI, which indicates a patient’s inflammatory and nutritional condition. The prognosis of HF patients is also significantly impacted by inflammation and nutritional state [18], [19], [20], [21], [22]. Therefore, we speculated that the CBR could predict the prognosis of all-cause mortality in patients with HF. The findings demonstrated that, as we expected, the CBR is a powerful and independent predictor of all-cause mortality across the spectrum of HF. This finding underscores the critical interplay between systemic inflammation and nutritional status in determining clinical outcomes in HF. Our results demonstrate that patients with a high CBR – indicative of heightened inflammatory activity and/or poor nutritional reserve – face a substantially elevated risk of mortality, independent of traditional risk factors and HF type.

CRP is an acute-phase reactant that is currently widely used as an indicator to assess systemic inflammation. Inflammation can lead to HF through the following mechanisms. First, systemic inflammation activates the sympathetic nervous system renin-angiotensin system, resulting in volume expansion, elevated peripheral vascular resistance, cardiomyocyte hypertrophy and apoptosis [23], 24]. Secondly, proinflammatory cytokines have the potential to produce endothelial dysfunction, pulmonary oedema, myocardial contractility inhibition, and left ventricular remodelling [25], 26]. In established HF, elevated CRP is related to cardiac decompensation as well as persistent harm to other organs, including reduced cardiac output and venous congestion, that can trigger the production of IL-6 [27]. This important cytokine activates CRP, which in turn can activate complement and amplify the inflammatory response by producing TNF-a, which may result in myocardial tissue damage or dysfunction [28]. The effects of CRP on organs other than the heart may also contribute to the progression of HF. Muscular wasting, renal dysfunction, and anaemia, which are common comorbidities in HF, are likely to result partly from inflammatory activation [29].

BMI is widely used as an indicator to evaluate the nutritional status of patients because it is easily accessible. BMI has been reported to be an independent risk factor for various cardiovascular diseases, including ventricular arrhythmias, atria, stroke, sudden cardiac death, acute coronary syndrome and HF [30], [31], [32]. Obesity leads to excessive deposition of epicardial adipose tissue, hypertrophy of the different chambers of the heart, impaired left ventricular diastolic filling/diastole, increased cardiac output leading to increased left ventricular stroke volume, increased myocardial oxygen consumption, and increased pulmonary arterial and capillary wedge pressures, which can lead to HF and result in downstream outcomes after the onset of HF [33]. Similarly, malnutrition can adversely affect the prognosis of patients with HF. Malnutrition can lead to decreased skeletal muscle strength and immune dysfunction in HF patients [34], 35]. In turn, decreased skeletal muscle strength can further exacerbate poor exercise tolerance as a result of lower cardiac output; immune dysfunction leads to frequent infections, which increases the risk of HF decompensation [36].

The CBR offers unique biological insights by integrating the pro-inflammatory state (reflected by CRP) and nutritional reserve (reflected by BMI) into a single metric. There is also an interaction between inflammation and nutritional status. Among the many metabolic impacts caused by inflammation are heightened insulin resistance and decreased appetite, which prevent nutrients from reaching cells [37], 38]. In HF, chronic inflammation and neurohormonal activation also encourage catabolism, which results in the breakdown of protein and fat tissue and, ultimatelycan progress beyond simple weight loss to a distinct entity known as cardiac cachexia [39]. Its pathophysiology is uniquely driven by the HF milieu. Persistent cardiac wall stress, gut hypo-perfusion, and concomitant comorbidities foster a chronic inflammatory state characterised by elevated, weight loss and cachexia [40], 41]. The interplay between systemic inflammation and nutritional status in HF cytokines such as IL-6 and TNF-α [42]. These mediators not only suppress appetite but also directly activate major proteolytic pathways in skeletal muscle, including the ubiquitin-proteasome system and autophagy, leading to accelerated muscle wasting [43]. This catabolic state is further exacerbated by the neurohormonal activation intrinsic to HF (e.g., elevated angiotensin II and aldosterone), which promotes insulin resistance and further catabolism [39], 44]. Therefore, cardiac cachexia represents an active, multi-systemic metabolic disorder, rather than a passive outcome of reduced caloric intake. Excess nutrition can trigger chronic inflammation, contribute to adipocyte hypertrophy, cause relative hypoxia of intracellular organelles, increase oxidative stress in the endoplasmic reticulum, and allow the release of pro-inflammatory factors into the bloodstream [45]. In addition, low BMI is often associated with malnutrition, which can mean the body is deficient in important nutrients such as proteins, vitamins and minerals. This can weaken the immune system, making the body less able to fight off pathogens, which can easily lead to infections, which in turn can lead to inflammation [46].

Given the pathophysiological overlap between a high CBR profile (elevated CRP and low BMI) and the hallmarks of cardiac cachexia – specifically, persistent systemic inflammation and a catabolic state leading to muscle wasting and weight loss – we hypothesize that an elevated CBR may serve as a simple, indirect clinical proxy for identifying HF patients who are at risk of developing or are already experiencing cardiac cachexia. The CBR integrates the two key driving forces of cachexia in HF: inflammation (CRP) and nutritional depletion/catabolism (reflected by low BMI). While direct assessment of muscle mass or specific cachexia diagnostic criteria provide a more definitive diagnosis, the CBR, derived from routinely available and inexpensive measures, could act as an early warning sign or a screening tool in busy clinical settings. This aligns with the finding in our study that the high CBR group exhibited significantly lower BMI and elevated CRP, a pattern indicative of an inflammation-driven catabolic state akin to cachexiaa [39], 42]. Future studies validating CBR against established cachexia criteria and its trajectory over time are warranted to confirm this utility.

Moreover, the interplay between fluid overload, inflammation, and cachexia represents a critical pathophysiological triad in advanced HF. Fluid overload exacerbates systemic venous congestion, which may increase gut permeability and promote endotoxin translocation, thereby amplifying inflammatory responses [47]. This persistent inflammation accelerates muscle catabolism through pathways such as ubiquitin-proteasome activation, leading to sarcopenia and cardiac cachexia [43]. In turn, cachexia further impairs cardiac and renal function, worsening fluid retention [42] – a vicious cycle that may be indirectly captured by an elevated CBR in the presence of low BMI (reflecting muscle loss rather than true leanness) and high CRP. Future studies should therefore aim to dissect these interactions using multimodal assessments that separate fluid status from true nutritional reserves.

To address the limitation of BMI in fluid-overloaded states, we propose that subsequent research incorporate low-cost, clinically feasible tools such as bioelectrical impedance analysis to differentiate extracellular water from fat-free mass [48]. Muscle ultrasound can also quantify muscle thickness and quality without radiation exposure [49]. Functional measures like handgrip strength and gait speed, which are less influenced by acute volume changes, could also serve as practical surrogates for muscle function and nutritional robustness [50]. Combining these with serial inflammatory biomarkers (e.g., IL-6, TNF-α) would allow a more nuanced understanding of how CBR reflects the balance between inflammation, nutrition, and fluid status in HF.

In summary, our findings demonstrate that the CBR is a robust and independent predictor of all-cause mortality in heart failure patients with different ejection fractions. The CBR combines CRP and BMI, thus can provide a more complete picture of the complex interplay between inflammation and nutrition than individual indicators. More importantly, the superior predictive performance of CBR over individual CRP and BMI, as evidenced by the larger AUC values, underscores its significant clinical application value. The CBR is calculated from routinely available and inexpensive laboratory (CRP) and anthropometric (BMI) data, making it a practical tool that can be easily integrated into existing clinical workflows. This simplicity positions the CBR as a potential bedside risk stratification indicator. In HF patients, the high CBR levels may suggest that the inflammatory process is causing significant damage to the body and that the patient’s nutritional reserve is insufficient to cope with the stress of the disease, which is often associated with a worse prognosis. As the CBR increased, the risk of all-cause mortality in patients also increased. This suggests that clinicians should be alert to the patient’s inflammatory and nutritional state and treat them when those factors are abnormal. For instance, patients identified as high-risk (e.g., CBR≥0.35 for all patients) based on admission data could be flagged for more intensive monitoring, earlier aggressive management, and comprehensive assessment of their inflammatory and nutritional status. This could include tailored nutritional support and anti-infective treatment or closer follow-up, which may potentially improve outcomes.

Furthermore, the CBR could be combined with established biomarkers and clinical parameters to create enhanced prognostic models, offering a more holistic view of the patient’s condition. The CBR reflects the association between inflammation and nutritional status. When paired with cardiac biomarkers like BNP (which shows cardiac stress), renal function markers (as HF impacts kidneys), and haemodynamic parameters (indicating heart function), it offers a more comprehensive view of the disease. This multi-factor approach improves prediction accuracy by creating a detailed risk assessment model and better stratifying patients. It helps to tailor treatment regimens and predict treatment response in the clinic.

Despite its promise, several limitations of the CBR must be considered for its clinical interpretation. Firstly, there may be some selection bias because it is an observational retrospective research. To validate the impact of the CBR on the prognosis of all-cause mortality in patients with HF, further prospective studies are needed. Secondly, as our study was a single-centre study, this may limit the generalization of the results to other populations. In the future, we could further develop multi-centre or international cohorts to validate in more diverse populations to improve generalizability. Thirdly, our study relied on BMI as a nutritional indicator, which may not accurately reflect true nutritional status due to confounding factors such as body composition, fluid overload, and cachexia. This could affect the interpretation of CBR, particularly in patients with oedema or wasting syndromes. Future research should incorporate direct assessments of muscle mass and fluid status, such as bioelectrical impedance analysis, to improve accuracy. Fourthly, our study did not account for the influence of medications (e.g., statins, anti-inflammatory drugs, diuretics) that may affect CRP levels or body weight. The impact of such medications on the CBR and its prognostic accuracy remains unclear and warrants investigation in future studies that systematically collect pharmacological data. Fifthly, although our study did not directly compare CBR with validated HF risk scores, such as MAGGIC or SHFM, due to data constraints, CBR offers a simple and cost-effective alternative that integrates both inflammatory and nutritional status. Future prospective studies should include these established scores to further validate CBR’s clinical utility and integration into multimodal risk stratification models. Finally, patients with HF who were in NYHA class III or IV were the primary subjects in this study, which may not represent the full range of heart failure severity. In the future, we will include HF patients with NYHA class I and II to make the results of this study more widely available.

## Conclusions

This study demonstrates that the CBR is an independent predictor of prognosis for all-cause mortality in HF patients with different ejection fractions. The CBR is roughly positively correlated with all-cause mortality in patients, suggesting that as the CBR increases, so does the risk of all-cause mortality in patients. Regardless of the type of HF, the CBR is a good predictor of a patient’s prognosis for all-cause mortality.
